# Differences in Anti-αvβ6 Integrin Antibody Expression between U.S. and Japanese Cohorts in Inflammatory Bowel Disease

**DOI:** 10.1093/ibd/izaf246

**Published:** 2025-11-22

**Authors:** Yoichi Kakuta, Dalin Li, Philip Debbas, Soshi Okazaki, Motoi Sawahashi, Shaohong Yang, Hideya Iwaki, Daisuke Okamoto, Hiroshi Nagai, Yusuke Shimoyama, Takeo Naito, Rintaro Moroi, Masatake Kuroha, Hisashi Shiga, Yoshitaka Kinouchi, Tsuyoshi Shirai, Hiroshi Fujii, Dermot P B McGovern, Atsushi Masamune

**Affiliations:** Division of Gastroenterology, Tohoku University Graduate School of Medicine, Sendai, 980-8574, Japan; F. Widjaja Inflammatory Bowel Disease Institute, Cedars-Sinai Medical Center, Los Angeles, CA, 90048, United States; F. Widjaja Inflammatory Bowel Disease Institute, Cedars-Sinai Medical Center, Los Angeles, CA, 90048, United States; Department of Rheumatology, Tohoku University Hospital, Sendai, 980-8574, Japan; Division of Gastroenterology, Tohoku University Graduate School of Medicine, Sendai, 980-8574, Japan; F. Widjaja Inflammatory Bowel Disease Institute, Cedars-Sinai Medical Center, Los Angeles, CA, 90048, United States; Division of Gastroenterology, Tohoku University Graduate School of Medicine, Sendai, 980-8574, Japan; Division of Gastroenterology, Tohoku University Graduate School of Medicine, Sendai, 980-8574, Japan; Division of Gastroenterology, Tohoku University Graduate School of Medicine, Sendai, 980-8574, Japan; Division of Gastroenterology, Tohoku University Graduate School of Medicine, Sendai, 980-8574, Japan; Division of Gastroenterology, Tohoku University Graduate School of Medicine, Sendai, 980-8574, Japan; Division of Gastroenterology, Tohoku University Graduate School of Medicine, Sendai, 980-8574, Japan; Division of Gastroenterology, Tohoku University Graduate School of Medicine, Sendai, 980-8574, Japan; Division of Gastroenterology, Tohoku University Graduate School of Medicine, Sendai, 980-8574, Japan; Student Healthcare Center, Institute for Excellence in Higher Education, Tohoku University, Sendai, 980-8576, Japan; Department of Rheumatology, Tohoku University Hospital, Sendai, 980-8574, Japan; Department of Rheumatology, Tohoku University Hospital, Sendai, 980-8574, Japan; F. Widjaja Inflammatory Bowel Disease Institute, Cedars-Sinai Medical Center, Los Angeles, CA, 90048, United States; Division of Gastroenterology, Tohoku University Graduate School of Medicine, Sendai, 980-8574, Japan

**Keywords:** anti-integrin αvβ6 antibody, anti-EPCR antibody, ulcerative colitis, Crohn’s disease

## Abstract

**Background:**

Inflammatory bowel diseases (IBDs), including ulcerative colitis (UC) and Crohn’s disease (CD), have complex pathologies requiring precise diagnostic tools. We evaluated the clinical utility of anti-integrin αvβ6 antibodies in diagnosing UC, focusing on differences between a U.S. cohort (self-reported White) and a Japanese cohort, and additionally assessed whether combining anti-αvβ6 with anti-EPCR improved diagnostic performance.

**Methods:**

Serum anti-αvβ6 antibody levels were measured in 1138 participants (514 in the U.S. cohort, 624 in the Japanese cohort), including 1093 IBD cases and 45 healthy control subjects. Positivity rates and titers were compared between cohorts, and associations with clinical subphenotypes and anti-EPCR were examined.

**Results:**

Anti-αvβ6 positivity was significantly higher in UC patients (85.4%) than in CD patients (16.4%) or control subjects (0%). Within UC, high positivity was observed across all disease extents, with only minor cohort differences. Longer disease duration was associated with lower positivity in both cohorts. In CD, the U.S. cohort showed higher positivity (23.4%) than the Japanese cohort (10.1%), particularly in colonic CD. Absence of ileal involvement, strictures, or perianal disease was associated with higher positivity. Anti-αvβ6 and anti-EPCR levels were strongly correlated, but their expression patterns differed in primary sclerosing cholangitis–associated IBD. Combining anti-αvβ6 and anti-EPCR improved UC diagnostic accuracy (area under the curve, 0.98; 95% confidence interval, 0.95-1.00) over either antibody alone (*P* = .00264).

**Conclusions:**

Anti-αvβ6 is a valuable biomarker for UC diagnosis. However, this study demonstrated differences in its behavior between U.S. and Japanese cohorts, particularly in CD. Cohort-informed interpretation and combined antibody testing may improve diagnostic precision and disease stratification in IBD.

Key Messages **What is already known?**

Anti-integrin αvβ6 and anti-EPCR antibodies have been proposed as potential diagnostic biomarkers for ulcerative colitis.

**What is new here?**

This study provides the first direct comparison of anti-αvβ6 antibody expression between a U.S. cohort (self-reported White) and a Japanese cohort, validates its diagnostic value using a commercial assay.

**How can this study help patient care?**

Interpretation of anti-αvβ6 results informed by differences between U.S. (self-reported White) and Japanese cohorts, together with combined antibody testing, may improve diagnostic precision and disease stratification in inflammatory bowel disease.

## Introduction

Inflammatory bowel diseases (IBDs) such as ulcerative colitis (UC) and Crohn’s disease (CD) have complex etiology and are characterized by chronic intestinal inflammation. Diagnosis is based on symptoms, blood tests, and particularly on assessing intestinal inflammation via endoscopy, imaging, and histology, which are crucial for differentiating CD and UC.^1–3^ A significant minority of IBD cases are defined as inflammatory bowel disease unclassified (IBDU) due to overlapping features of UC and CD.^4^^,^^5^ Even in clear UC or CD diagnoses, clinical heterogeneity exists, including variable treatment responses. Thus, developing biomarkers linked to disease etiology and useful for patient stratification is essential.

In recent years, 2 new autoantibodies have been reported to be useful for the diagnosis of UC. We previously identified high positivity rates for anti-EPCR in Japanese patients with UC,^6^ and found that this antibody is associated with various disease subphenotypes, findings confirmed in UC cases among those of a U.S. cohort (self-reported White).^7^ In 2020, Kuwada et al^8^ first reported anti-αvβ6 as an autoantibody in Japanese UC cases. Subsequently, such association was also reported in U.S. and European-origin cases, while this antibody’s potential as a therapeutic target was also investigated.^9–12^

These two new diagnostic biomarkers are anticipated to have clinical applications. In particular, anti-αvβ6 measurement kits have been developed, facilitating the measurement of antibody titers in the blood. However, both antibodies were discovered in UC patients of the Japanese cohort, and although several studies have been conducted in multiethnic populations,^13^ no direct comparisons have been made between the Japanese and U.S. cohorts. Moreover, the link between anti-αvβ6 titers and clinical phenotypes remains unclear, although higher levels have been reported in IBD cases with primary sclerosing cholangitis (PSC).^14^ Because PSC often co-occurs with atypical IBD, understanding how these two antibodies contribute to PSC-associated IBD is important.^15–17^

In this study, we evaluated the clinical usefulness of anti-αvβ6 measured by a commercially available ELISA kit for UC diagnosis and examined cohort differences in positivity rates. Additionally, we analyzed the clinical phenotypes of UC and CD associated with anti-αvβ6 and investigated its relationship with anti-EPCR.

## Methods

### Participants

Serum samples from 1138 participants, including 1093 cases and 45 healthy control subjects, were analyzed in this study (Figure S1). Participants consisted of 514 individuals from a U.S. cohort (all self-reported White) recruited at Cedars-Sinai Medical Center and 624 individuals from a Japanese cohort recruited at Tohoku University Hospital and 8 affiliated hospitals in Japan. Comparisons of age and sex by cohort and disease group are summarized in Table S1. Of the 1093 cases, 1041 had IBD and 52 had other intestinal diseases. These 52 cases were grouped as “Others” and included diseases such as intestinal Behçet’s disease, microscopic colitis, diverticular disease, and colorectal cancer. Of the IBD cases (Table S2), 456 participants had UC (5 UC cases were recruited after total colectomy), 566 had CD, and 19 carried a diagnosis of IBDU. Control subjects were healthy volunteers recruited independently at each institution (Cedars-Sinai Medical Center for the U.S. cohort and Tohoku University and affiliated hospitals for the Japanese cohort). No age- or sex-matching procedures were applied.

This study was approved by the Ethics Committees of Tohoku University School of Medicine and Cedars-Sinai Medical Center. The study was performed in accordance with the ethical standards laid down in the 1964 Declaration of Helsinki and its later amendments. All Japanese samples and clinical information were collected through the IBD-MOCHA Study (Approval Nos. 2021-1-1244, 2023-1-106) and the MIRAI Study (Approval No. 2022-1-249). All samples from Cedars-Sinai were collected through the MIRIAD database (Approval No. 3358). All participants provided written informed consent.

### Measurement of serum antibody titers and cutoff values

Serum IgG against integrin αvβ6 was measured using Anti-Integrin αvβ6 ELISA Kit (Medical & Biological Laboratories) following the manufacturer’s protocol. The cutoff values for anti-αvβ6 were based on the manufacturer’s recommendations: those of ≤2.69 U/mL were considered negative, and those of >2.69 U/mL were considered positive. For anti-EPCR, we used data from 265 samples for which measurement results for the same samples in a previous study were available.^7^ In the previous study, the binding capacities of anti-EPCR in serum were measured by cell-based assay using flow cytometry. The cutoff value for anti-EPCR was set at 21.9, in accordance with the previous study.

### Collection of clinical information

IBDs, including CD, UC, and IBDU, were diagnosed based on clinical symptoms and endoscopic, radiographic, and histological findings, in accordance with conventional criteria.^18^ Clinical information was collected from medical records, including age, sex, disease type, age at diagnosis, extent of disease, extraintestinal complications, and IBD-associated surgical history.

### Statistical analysis

For titers of anti-αvβ6 by disease and clinical subtype, the Mann-Whitney *U* test was used for 2-group comparisons, the Kruskal-Wallis test was used for comparisons of 3 or more groups, and the chi-square test was used for comparisons of antibody positivity rates. For the chi-square test, odds ratios (ORs) and their 95% confidence intervals (CIs) were computed. Regression analysis was used to determine the relationship between continuous variables and antibody titers, and Spearman’s rank correlation coefficient was calculated. Items with *P* values of <.05 in univariate analyses were evaluated in multivariate analysis. To account for multiple comparisons in univariable analyses, false discovery rate (FDR) adjustment was applied using the Benjamini-Hochberg method, and results are reported as q values where appropriate. In the main text, q < 0.05 was considered statistically significant. We calculated the continuous net reclassification improvement (NRI) and integrated discrimination improvement (IDI) to compare the performance of the anti-αvβ6–only and anti-αvβ6 + anti-EPCR models. Both metrics were derived from logistic regression-based predicted probabilities of the event. We used bootstrap resampling (1000 iterations) to obtain 95% CIs for the NRI and IDI. For comparisons of area under the curve (AUC) values, we used DeLong’s test.

All statistical analyses were performed on Python 3.10.8 (Python Software Foundation) using pandas, scikit-learn and related libraries, and matplotlib and seaborn for plotting. For analyses where FDR adjustment was not applied (eg, multivariate regression, NRI/IDI, receiver-operating characteristic [ROC] comparisons), a *P* value of <.05 was considered statistically significant.

## Results

### Anti-αvβ6 positivity and titers in UC

**Figure 1.**
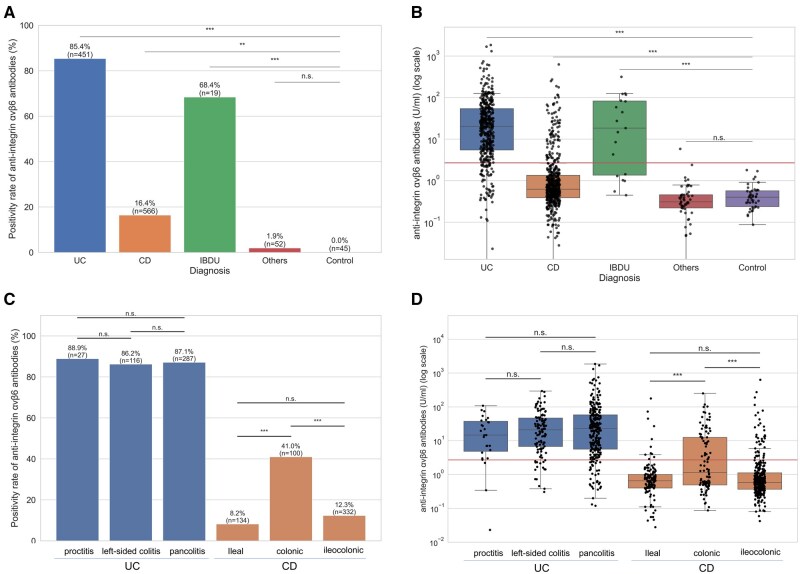
Titers and positivity rates of anti-αvβ6 by disease groups. (A) Positivity rates of anti-αvβ6 by disease group. (B) Scatter and box plots indicating anti-αvβ6 titers by disease group. (C) Positivity rates of anti-αvβ6 by disease extent in ulcerative colitis (UC) and by disease location in Crohn’s disease (CD). (D) Scatter and box plots indicating anti-αvβ6 titers by disease extent in UC and by disease location in CD. In panels B and D, the red line indicates the cutoff titer, and the vertical axis is logarithmic. Statistical significance is indicated by asterisks based on q values (false discovery rate–adjusted *P* values by Benjamini-Hochberg correction): *q < 0.05, **q < 0.01, ***q < 0.001. Raw *P* values and corresponding q values are provided in Tables S3. IBDU, inflammatory bowel disease unclassified; n.s., not significant.

In the overall cohort, anti-αvβ6 positivity was markedly higher in UC patients (85.4%) than in CD patients (16.4%), IBDU patients (68.4%), or control subjects (0%) (q < 0.001, FDR adjusted) (Figure 1A). Only 1 positive case was observed among intestinal diseases other than IBD (microscopic enterocolitis, 1.9%). In IBDU patients, positivity rates were also higher than in control subjects, though the number of cases was small. Antibody titers showed similar trends, being significantly elevated in UC patients and, to a lesser extent, in CD and IBDU patients compared with control subjects, but consistently lower in CD patients than in UC patients (Figure 1B; Table S3). Analyses of postoperative UC are shown in Figure S2, indicating lower positivity (40%) and titers than in nonoperated UC, though numbers were very small.

### Subtype and clinical factors associated with anti-αvβ6 positivity

The differences in anti-αvβ6 antibody positivity and titers at baseline among the disease subphenotypes were examined (Table 1; Table S4). In UC, positivity rates were uniformly high—88.9% in E1, 86.2% in E2, and 87.1% in E3—with no significant differences between subtypes (Figure 1C). Titers showed similar patterns, with no significant differences by extent (Figure 1D). In CD, colonic disease (L2) had markedly higher positivity (41.0%) than ileal (L1, 8.2%) or ileocolonic (L3, 12.3%) disease (q < 0.001 for both comparisons, FDR adjusted) (Figure 1C); titers were likewise higher in L2 than in L1 or L3 (q < 0.001), with no difference between L1 and L3 (Figure 1D). Both UC and CD subphenotypes showed higher positivity than non-IBD control subjects (q < 0.001).

**Table 1.** Association of anti-integrin αvβ6 antibody with disease subphenotypes.

Phenotype	n	Titer of anti-αvβ6 (U/mL)	Positivity of anti-αvβ6			
			Negative (n = 66)	Positive (n = 385)	*P* value	FDR
Ulcerative colitis						
Cohort					**.0460^a^**	0.107
U.S. (self-reported White)	212	19.1 (3.93-44.8)	39	173 (81.6%)		
Japanese	239	23.0 (6.88-56.9)	27	212 (88.7%)		
Sex					.192	0.336
Female	216	17.8 (4.61-54.4)	37	179 (82.9%)		
Male	235	24.4 (6.00-51.0)	29	206 (87.7%)		
Age, y	451	20.2 (5.48-54.2)	51.5 (38.3-63.8)	42.0 (30.0-56.0)	**<.001^a^**	**<0.001^a^**
Age at diagnosis, y	418	21.6 (5.91-55.2)	29.5 (22.8-38.8)	28.5 (20.0-40.0)	.425	0.595
Disease duration	418	21.6 (5.91-55.2)	14.5 (9.0, 28.0)	9.0 (4.0, 16.0)	**<.001^a^**	**<0.001^a^**
Disease extent					.927	0.927
E1 (proctitis)	27	14.7 (4.86-37.4)	3	24 (88.9%)		
E2 (left-sided colitis)	116	20.9 (6.76-46.7)	16	100 (86.2%)		
E3 (pancolitis)	287	22.9 (5.63-57.9)	37	250 (87.1%)		
Colectomy^b^					.837	0.927
Yes	6	50.2 (19.6-118)	0	6 (100.0%)		
No	232	22.9 (6.92-56.5)	26	206 (88.8%)		
Crohn’s disease						
Cohort					**<.001^a^**	**<0.001^a^**
U.S. (self-reported White)	269	0.785 (0.337-2.23)	206	63 (23.4%)		
Japanese	297	0.559 (0.398-0.965)	267	30 (10.1%)		
Sex					.0672	0.0874
Female	217	0.703 (0.398-1.85)	300	49 (14.0%)		
Male	349	0.604 (0.385-1.16)	173	44 (20.3%)		
Age, y		0.624 (0.389-1.34)	44.0 (34.0-55.0)	41.0 (31.0-53.0)	.172	0.203
Age at diagnosis, y	551	0.624 (0.389-1.298)	23.0 (18.0-32.0)	25.0 (18.0-36.0)	.570	0.570
Disease location					**<.001^a^**	**<0.001^a^**
L1 (ileal)	134	0.653 (0.398-1.00)	123	11 (8.2%)		
L2 (colonic)	100	1.149 (0.491-12.5)	59	41 (41.0%)		
L3 (ileocolonic)	332	0.582 (0.366-1.12)	291	41 (12.3%)		
Upper gastrointestinal disease					**<.001^a^**	**0.0018^a^**
No	372	0.677 (0.384-1.80)	294	78 (21.0%)		
Yes	132	0.569 (0.390-0.921)	122	10 (7.6%)		
Small bowel involvement					**<.001^a^**	**<0.001^a^**
No	100	1.15 (0.491-12.5)	59	41 (41.0%)		
Yes	466	0.600 (0.377-1.10)	414	52 (11.2%)		
Colonic involvement					**.00501^a^**	**0.0081^a^**
No	134	0.653(0.398-1.00)	123	11 (8.2%)		
Yes	432	0.614 (0.385-1.54)	350	82 (19.0%)		
Disease behavior					**<.001^a^**	**<0.001^a^**
B1	213	0.735 (0.403-3.61)	156	57 (26.8%)		
B2	170	0.531 (0.360-0.949)	155	15 (8.8%)		
B3	170	0.642 (0.389-1.23)	151	19 (11.2%)		
Fistula					**.0362^a^**	0.0523
No	384	0.614 (0.391-1.45)	312	72 (18.8%)		
Yes	170	0.642 (0.390-1.23)	151	19 (11.2%)		
Stenosis					**<.001^a^**	**<0.001^a^**
No	251	0.697 (0.398-2.62)	189	62 (24.7%)		
Yes	306	0.582 (0.378-1.08)	277	29 (9.5%)		
Perianal disease					**.00354^a^**	**0.0066^a^**
No	320	0.657 (0.385-1.87)	254	66 (20.6%)		
Yes	244	0.605 (0.390-1.05)	217	27 (11.1%)		
Intestinal resection					.242	0.262
No	95	0.553 (0.370-0.945)	82	13 (13.7%)		
Yes	200	0.564 (0.404-0.980)	183	17 (8.5%)		

Values are median (interquartile range), n, or n (%).

a p < 0.05 or FDR < 0.05

b Colectomy after serum sampling.

Abbreviation: FDR, false discovery rate.

**Figure 2.**
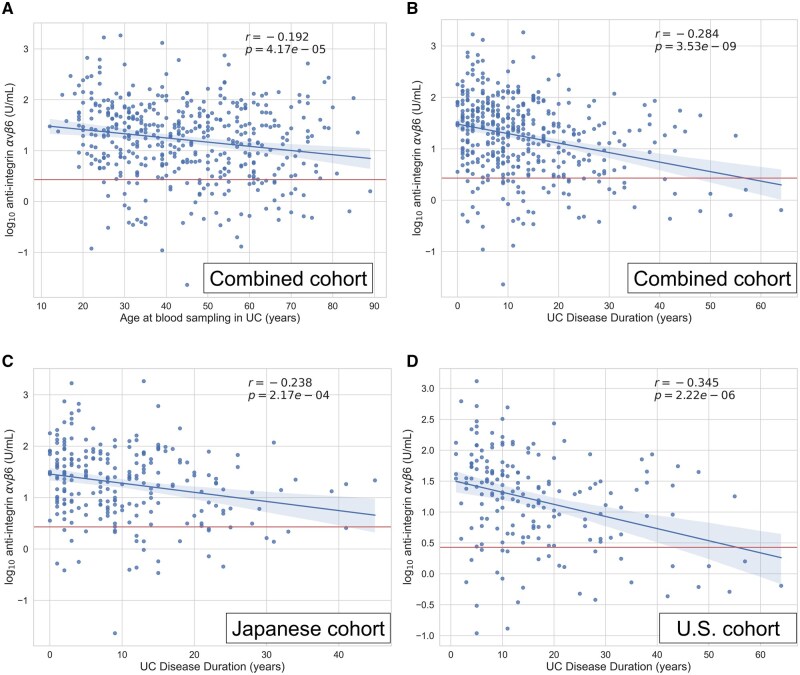
Associations of age at blood sampling and disease duration with anti-αvβ6 titers in ulcerative colitis (UC). Scatter plots illustrate the correlation between anti-αvβ6 titers and (A) age at blood sampling or (B-D) disease duration in UC. Panels A and B show the combined cohort, while panels C and D present analyses stratified by U.S. cohort (self-reported White) and Japanese cohort. The values for r and *P* are from Spearman’s correlation; *P* values are shown in scientific notation when very small (<.001). The blue lines indicate linear regression fit with 95% confidence intervals. The red horizontal line marks the assay’s cutoff for anti-αvβ6 positivity. Corresponding false discovery rate–adjusted q values from linear models are reported in Table S4.

In UC, both age at blood sampling and disease duration demonstrated significant negative associations with anti-αvβ6 positivity (Table 1) and titers (Table S4). Specifically, anti-αvβ6 titers were lower in older subjects (r = –0.192; *P* < .001), and even lower in those with longer disease duration (r = –0.284; *P* < .001) (Figure 2A and B). When analyzed separately in the Japanese cohort (Figure 2C) and the U.S. cohort (Figure 2D), this pattern remained consistent. Notably, these correlations with age and disease duration were not observed in CD (Figure S3A-D).

In CD, higher antibody positivity was associated with the absence of small bowel lesions, upper gastrointestinal lesions, strictures, or perianal disease (Table 1). Conversely, positivity rates were lower in CD cases with these lesions or behaviors. However, some of these associations did not remain significant in the multivariate model, likely due to co-associations among clinical factors (eg, ileal disease being more frequently accompanied by characteristic CD lesions).

### Differences between U.S. and Japanese cohorts

**Figure 3.**
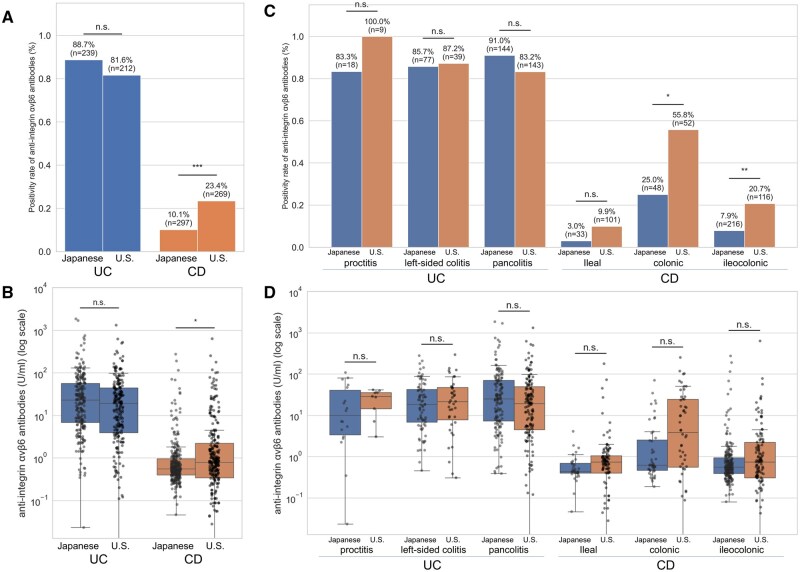
Differences in titers and positivity rates of anti-αvβ6 between the U.S. and Japanese cohorts. (A) Positivity rates of anti-αvβ6 in ulcerative colitis (UC) and Crohn’s disease (CD) by cohort. (B) Scatter and box plots of anti-αvβ6 titers in UC and CD by cohort. (C) Positivity rates of anti-αvβ6 in UC by disease extent and in CD by disease location, stratified by cohort. (D) Scatter and box plots of anti-αvβ6 titers in UC by disease extent and in CD by disease location, stratified by cohort. The red line in scatter plots indicates the cutoff titer, and vertical axes are logarithmic. Statistical significance is indicated by asterisks based on q values (false discovery rate–adjusted *P* values by Benjamini-Hochberg correction): *q < 0.05, **q < 0.01, ***q < 0.001. Raw *P* values and corresponding q values are provided in Table S5. n.s., not significant.

**Figure 4.**
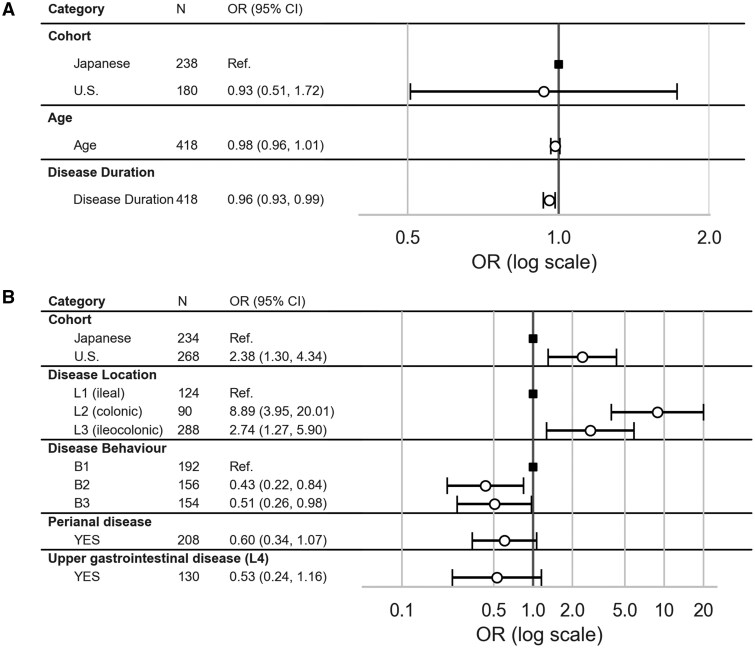
Forest plots of logistic regression analysis for factors associated with anti-αvβ6 positivity. Results of multivariate analysis on factors associated with antibody positivity in (A) ulcerative colitis (A) and (B) Crohn’s disease. The white circles represent the odds ratios (ORs), and the bars indicate their 95% confidence intervals (CIs).

Positivity and titers of anti-αvβ6 were compared between the U.S. and Japanese cohorts (Figure 3A and B). The patient backgrounds for each cohort are summarized in Table S5. In UC, positivity was slightly lower in the U.S. cohort (81.6%) compared with the Japanese cohort (88.7%), but this difference did not reach significance (q = 0.081). Across UC subtypes, positivity remained high in both cohorts, with pancolitis showing a tendency toward lower positivity in the U.S. cohort (83.2% vs 91.0%) (Figure 3C). In multivariate analysis including cohort, age, and disease duration, disease duration was the only independent factor associated with antibody positivity in UC (Figure 4A; Table S6), while age and cohort were not significant.

In contrast, for CD, the positivity rate was significantly higher in the U.S. cohort (23.4%) compared with the Japanese cohort (10.1%; q < 0.001) (Figure 3A). When stratified by disease location, positivity remained consistently higher in the U.S. cohort, most notably in colonic CD (L2: 55.8% vs 25.0%; q = 0.010) and also in ileocolonic CD (L3: 20.7% vs 7.9%; q = 0.008) (Figure 3C). Titers showed parallel trends, being generally higher in the U.S. cohort across all CD subtypes (Figure 3D). In multivariate analysis, both cohort and disease location were independently associated with antibody positivity (Figure 4B; Table S6).

### Correlation of anti-αvβ6 with anti-EPCR

**Figure 5.**
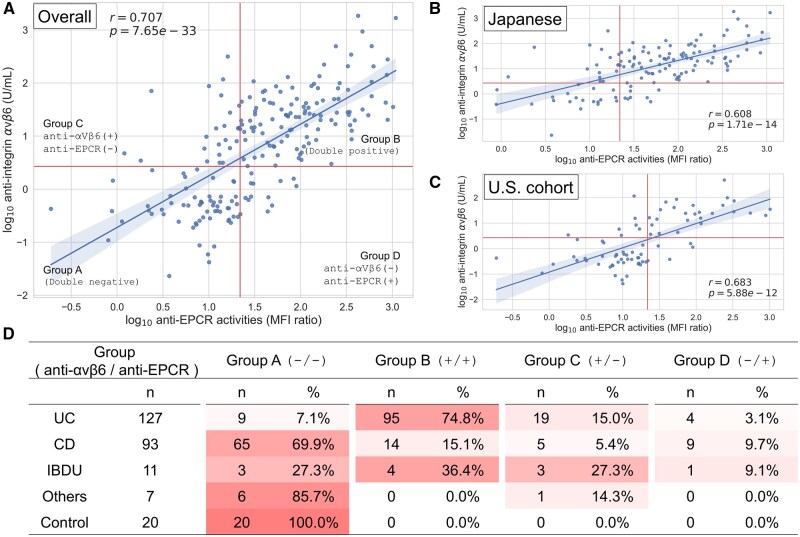
Association and combination of anti-αvβ6 with anti-EPCR. (A-C) Scatter plots illustrating the correlation between anti-αvβ6 and anti-EPCR titers. The correlation coefficients (r) and *P* values are based on Spearman’s correlation test. A straight line represents the regression fit, and the red lines indicate the cutoff values for each antibody. (A) Displays the entire cohort, with cases divided into 4 groups according to cutoff values: both negative (group A), both positive (group B), anti-αvβ6 only (group C), and anti-EPCR only (group D). (B, C) The plots for the Japanese cohort and the U.S. cohorts, respectively. (D) Illustrates the distribution of cases across the 4 groups for each disease, in which the intensity of the red color reflects the frequency of cases in each group, with darker red indicating a higher number of cases. CD, Crohn’s disease; IBDU, inflammatory bowel disease unclassified; MFI, mean fluorescence intensity; UC, ulcerative colitis.

Anti-αvβ6 titers showed a strong positive correlation with anti-EPCR titers across the entire cohort (r = 0.707; *P* = 7.65 × 10^−33^) (Figure 5A). This relationship remained significant in both the U.S. and Japanese cohorts (Figure 5B and C). When classified by antibody positivity, most UC patients were double positive for both anti-αvβ6 and anti-EPCR, while nearly all control subjects and most CD patients were double negative. In contrast, IBDU cases showed a heterogeneous distribution without a clear trend (Figure 5D; Table S7).

### Diagnostic performance of anti-αvβ6 for UC

**Figure 6.**
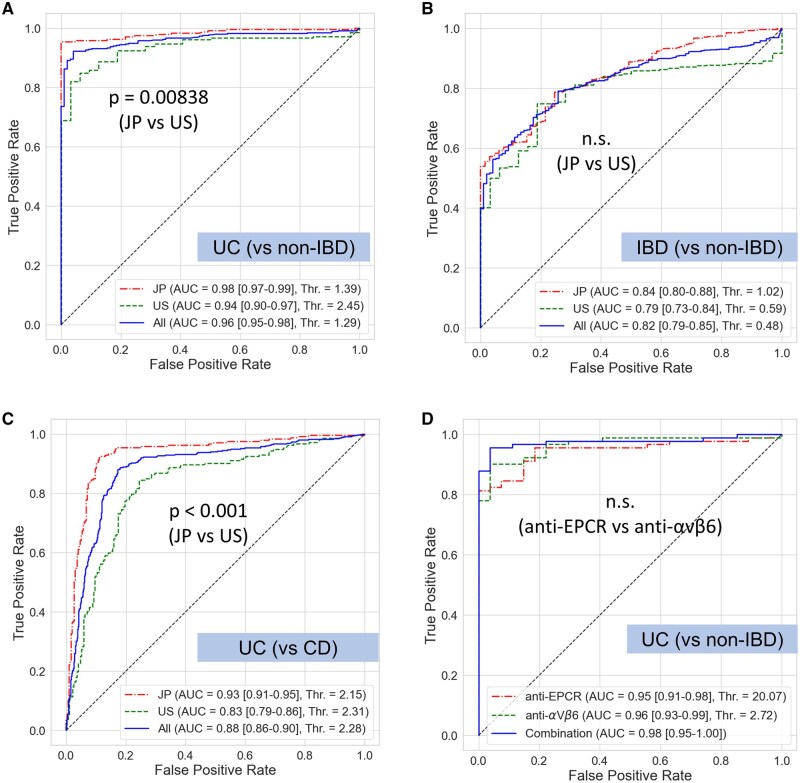
Receiver-operating characteristic analysis of anti-αvβ6 antibody. Receiver-operating characteristic analysis was performed on the diagnostic performance of anti-αvβ6 antibody. The best thresholds were calculated using Youden’s index, and area under the curve (AUC) comparisons were performed by DeLong’s test. (A) Diagnosis of ulcerative colitis (UC) vs non–inflammatory bowel disease (IBD) cases. (B) Diagnosis of IBD vs non-IBD. (C) Differential diagnosis between UC and Crohn’s disease (CD). (D) Diagnosis of UC using anti-EPCR, anti-αvβ6, and their combination. AUC values are shown with 95% confidence intervals The U.S. cohort (US) was self-reported White. The Threshold was the best cutoff value. JP, Japanese cohort; Thr., threshold.

ROC analysis was performed to evaluate diagnostic performance for various outcomes (Table S8). Identification of UC from non-IBD (control subjects or cases with intestinal diseases other than IBD) had high sensitivity of 0.92 and specificity of 0.96 when the cutoff value was 1.29 U/mL, with an AUC of 0.96 in the whole cohort (Figure 6A). The AUC was significantly higher in the Japanese cohort (0.98; 95% CI, 0.97-0.99) than in the U.S. cohort (0.94; 95% CI, 0.90-0.97; *P* = .00838, DeLong’s test). For differentiating IBD from non-IBD, the AUC was 0.82 (95% CI, 0.79-0.85) (Figure 6B), and UC and CD could be distinguished with an AUC of 0.88 (95% CI, 0.86-0.90) (Figure 6C).

For cases with available anti-EPCR data, AUC values for UC versus non-IBD were 0.95 (95% CI, 0.91-0.98) for anti-EPCR, 0.96 (95% CI, 0.93-0.99) for anti-αvβ6, and 0.98 (95% CI, 0.95-1.00) for the combination of both (Figure 6D). The combined model showed a statistically significant improvement in diagnostic ability compared with anti-αvβ6 alone (NRI = 0.827; 95% CI, 0.264-1.40; *P* = .00264; IDI = 0.127; 95% CI, 0.037-0.271; *P* = .0221). Anti-αvβ6 also retained high diagnostic performance in older-onset UC (≥60 years), with details provided in Table S9 and Figure S4.

## Discussion

To the best of our knowledge, this is the largest analysis to date studying the role of anti-αvβ6 for diagnosing UC and the first to directly compare positivity rates between U.S. and Japanese cohorts. In the first report by Kuwada et al,^8^ anti-αvβ6 showed a high positive rate of more than 95% in Japanese cases with UC, while the rate was about 10% in CD and almost 0% in other diseases and control subjects. Subsequently, a Swedish report, although with a limited number of cases, showed a positive rate of 76.3% for UC and 21.1% for CD, indicating a slight decrease in UC and an increase in positivity in CD compared with the separate Japanese study.^9^ An analysis of UC in the United States showed positivity of 85.5% and 70.2% in 2 cohorts, which were still high but were lower than in the first Japanese report.^11^ These findings suggested that cohort background may influence anti-αvβ6 positivity, but methodological differences in measurement and cutoff values across studies make direct comparisons challenging. Our study, using a large cohort, overcomes these issues and provides a robust comparison between cohorts.

Subphenotypes of Japanese CD differ from “Western” CD. In Japan, CD is more commonly reported in males, with ileocolonic type, complications of upper gastrointestinal lesions (L4), and perianal disease phenotypes being more common than in reports from predominantly European-origin populations.^19^ To account for these differences, analyses were conducted by disease type and multivariate analyses including clinical phenotypes. These analyses indicate that the higher anti-αvβ6 in the U.S. cohort CD cases is not due to differences in clinical phenotypes, but rather suggests that cohort background is an independent risk factor (in contrast to UC). Factors beyond clinical phenotype, such as genetic background or environmental exposures, may therefore underlie the higher antibody positivity observed in U.S. CD. Such findings supports the idea of subtle etiological differences between CD in Japanese and U.S. patients, as previously suggested in genetic studies.^20–23^ Collectively, these observations underscore the etiological heterogeneity underpinning the clinical diagnosis of CD.^24^

For UC, this study confirmed a slight tendency for lower anti-αvβ6 titers in the U.S. cohort, although levels remained high across cohorts. Thus, this antibody remains a useful UC biomarker regardless of cohort, although its significance in CD may vary. While UC consistently shows high titers, limiting its use for stratification, CD exhibits variability. In CD, the antibody correlates with fewer small bowel, upper gastrointestinal, anal, and stenotic lesions—features important for distinguishing it from UC. This suggests that CD with anti-αvβ6 is less “Crohn’s-like,” is more closely associated with colonic inflammation, and may represent a different IBD type or a UC-like CD phenotype.

In UC, anti-αvβ6 levels were lower in older age subjects and in those with longer disease duration but were not associated with age at diagnosis, suggesting disease duration as the key factor. This pattern raises the possibility that antibody levels decline over time, potentially reflecting age-related immune modulation or cumulative effects of disease chronicity, although longitudinal data will be required to confirm this trend. Shorter disease duration correlated with higher antibody titers, supporting dynamic changes over time. Previous studies have shown that these antibodies increase before disease onset and decrease gradually thereafter.^11^^,^^25^ Whether these changes are driven by therapeutic effects or natural disease progression remains unclear. The decrease with longer disease duration suggests that this study may have underestimated anti-αvβ6’s diagnostic accuracy at onset. Thus, future prospective studies should focus on newly diagnosed cases to better evaluate the diagnostic utility of this biomarker.

We previously reported anti-EPCR as another UC-associated autoantibody. The two antibodies share high UC positivity and links to colonic CD. The strong correlation observed in this study suggests that the two antibodies capture overlapping but distinct aspects of UC immunopathology, while their divergent expression in IBDU may help explain heterogeneity in disease classification. This study newly demonstrates the strong positive correlation in their titers. However, these two antibodies behave somewhat differently in IBDU cases, especially in those with IBD complicated with PSC and UC-like inflammation in the right colon, where the positivity of anti-αvβ6 and that of anti-EPCR do not match.^14^ It should be noted that these observations are limited to IBDU, particularly those with PSC, and we did not assess the broader impact of extraintestinal manifestations on antibody levels in UC or CD. Additionally, anti-EPCR is a marker for Takayasu arteritis, but the anti-αvβ6 positivity rate is low in Takayasu arteritis without UC.^26^ These findings suggest that the two antibodies may reflect different underlying pathophysiology. Further clarification of the mechanism of positivity of each antibody may help to elucidate the pathophysiology of these types of IBD. Moreover, our analysis demonstrated the diagnostic improvement when combining anti-αvβ6 with anti-EPCR antibodies. While the NRI and IDI analyses suggested additive value, caution is warranted in interpreting these metrics in smaller or imbalanced datasets.^27^ Future research should refine multimarker panels for IBD diagnostics.

Despite providing new insights, this study has several limitations. Although this is the largest comparative analysis of anti-αvβ6 across cohorts in IBD to date, the overall sample size remains limited, especially for control subjects. In addition, the positivity cutoff was based on the manufacturer’s recommendation, but whether this is optimal across different populations is unclear. Furthermore, the U.S. cohort was restricted to self-reported White individuals, and no generational background data were available, meaning that our study does not capture pan-ancestral diversity. Moreover, while disease activity data were available in the Japanese cohort, comparable data were not collected for the U.S. cohort. Because the primary objective of this study was to compare antibody profiles between the two cohorts, we excluded activity- or treatment-related analyses to avoid bias introduced by uneven data availability. Nevertheless, the lack of harmonized information on disease activity and treatment across cohorts may still influence the interpretation of our findings, and should be addressed in future studies. Future studies with larger sample sizes and diverse genetic backgrounds will be essential to refine this threshold and further validate the clinical utility of anti-αvβ6.

In conclusion, anti-αvβ6 is a highly sensitive and specific biomarker for the diagnosis of UC. Furthermore, we have shown that, in IBD—particularly in CD—anti-αvβ6 exhibits different behavior between U.S. (self-reported White) and Japanese cohorts. In the future, this antibody may be clinically applied as a biomarker for diagnosing IBD and, in combination with anti-EPCR antibodies, it may also have potential for substratification of IBD. Further studies should examine its role across diverse populations and refine cutoff values to determine its clinical utility.

## Supplementary Material

izaf246_Supplementary_Data

## Data Availability

The data supporting the findings of this study are available from the corresponding author, Y.Kakuta, upon reasonable request. These data are not publicly accessible due to privacy or ethical restrictions, as they contain sensitive information that could potentially compromise the confidentiality of the research participants.
