# Supplementary material for: Differences in Anti-αvβ6 Integrin Antibody Expression between U.S. and Japanese Cohorts in Inflammatory Bowel Disease
Source: Inflamm Bowel Dis. 2025 Nov 22;32(1):130–40. doi: 10.1093/ibd/izaf246 (PMC12759057; doi:10.1093/ibd/izaf246)
Supplement: izaf246_Supplementary_Data [file izaf246_supplementary_data.pdf]

Supplementary Materials

Differences in Anti-αvβ6 Integrin Antibody Expression between U.S. and Japanese Cohorts in Inflammatory Bowel Disease

Supplementary Figure

Supplementary Figure S1: Breakdown of the study cohort.

Supplementary Figure S2: Titers and positivity rates of anti-αvβ6 of postoperative UC

Supplementary Figure S3: Associations of age at blood sampling and disease duration with anti-αvβ6 titers in CD

Supplementary Figure S4: ROC curve for anti-αvβ6 in older-onset UC (age at diagnosis ≥60 years) versus non-IBD

Supplementary Tables

Supplementary Table S1. Basic characteristics of the study population

Supplementary Table S2. Breakdown of other intestinal diseases and positivity rates of anti-integrin αvβ6 antibody.

Supplementary Table S3. Titers and positivity rates of anti-integrin αvβ6 antibody.

Supplementary Table S4. Association of anti-integrin αvβ6 antibody titer with disease sub-phenotypes

Supplementary Table S5. Comparison of patient backgrounds and anti-αvβ6 by ethnicity

Supplementary Table S6. Multivariate analysis for association of anti-integrin αvβ6 antibody with disease sub-phenotypes

Supplementary Table S7. Phenotypes and two autoantibodies in Japanese patients with IBDU.

Supplementary Table S8. Results of the ROC analysis for anti-αvβ6 antibodies

Supplementary Table S9. Diagnostic performance of anti-αvβ6 in older-onset UC (age at diagnosis ≥60 years) vs non-IBD

Supplementary Figure

Supplementary Figure S1: Breakdown of the study cohort.

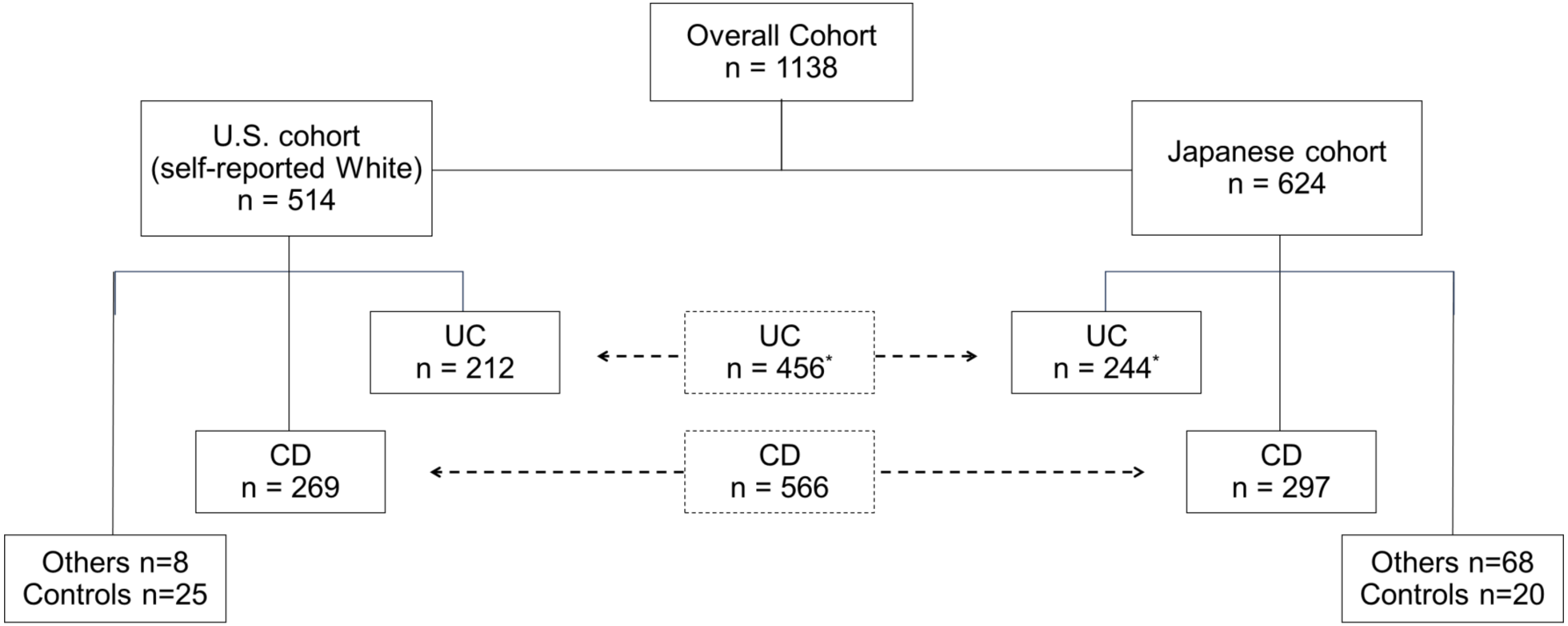

The diagram illustrates the breakdown of the overall cohort (N=1138) into the U.S. cohort (self-reported White, N=514) and the Japanese cohort (N=624).

Each cohort is further divided into categories: UC, CD, other diseases, and controls. *Five postoperative UC cases were included.

UC, ulcerative colitis; CD, Crohn's disease

Supplementary Figure S2: Titers and positivity rates of anti-αvβ6 of postoperative UC

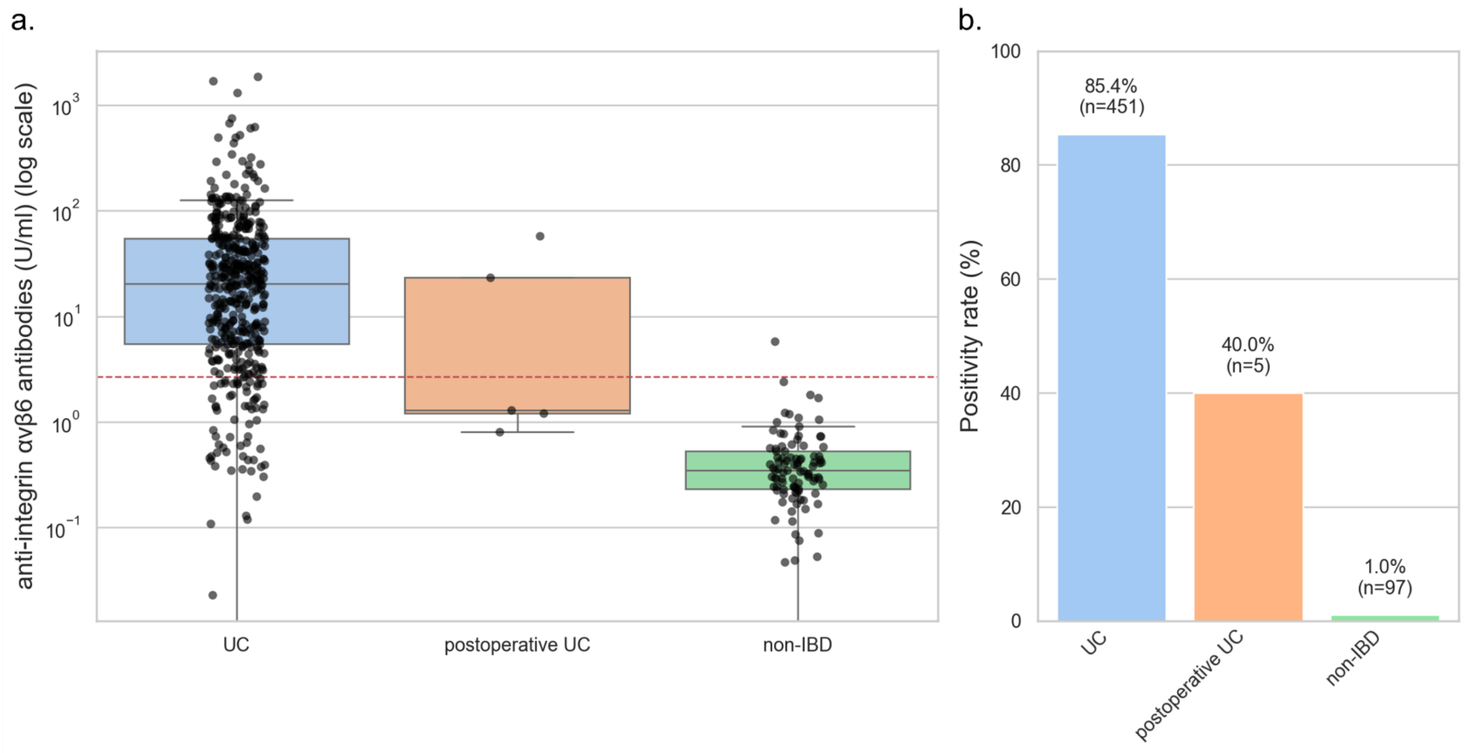

(a) Scatter and box plots showing anti-αvβ6 titers in postoperative UC compared with non-operated UC and non-IBD controls. The red line indicates the cut-off value, and the vertical axis is logarithmic. (b) Positivity rates of anti-αvβ6 in postoperative UC compared with non-operated UC and non-IBD controls. anti-αvβ6, anti-integrin αvβ6 antibody; UC, ulcerative colitis.

Supplementary Figure S3: Associations of age at blood sampling and disease duration with anti-αvβ6 titers in CD

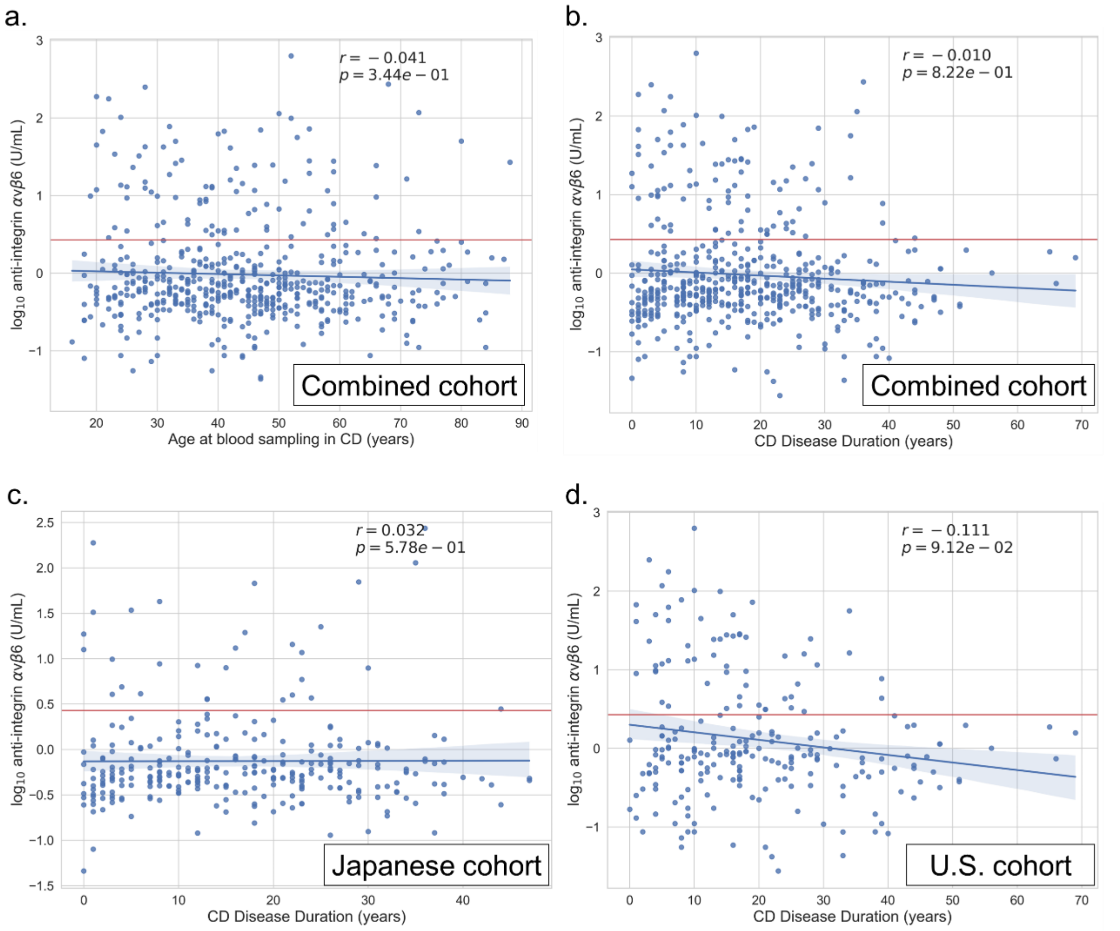

Scatter plots illustrate the correlation between anti-αvβ6 titers and (a) age at blood sampling or (b–d) disease duration in CD. Panels (a) and (b) show the combined cohorts, while panels (c) and (d) present the Japanese cohort (c), and the U.S. cohort (d). The values for r and p are from Spearman's correlation. The blue lines indicate linear regression fit with 95% confidence intervals. The red horizontal line marks the assay's cutoff for anti-αvβ6 positivity. CD, Crohn's disease;

Supplementary Figure S4: ROC curve for anti-αvβ6 in older-onset UC (age at diagnosis ≥60 years) versus non-IBD

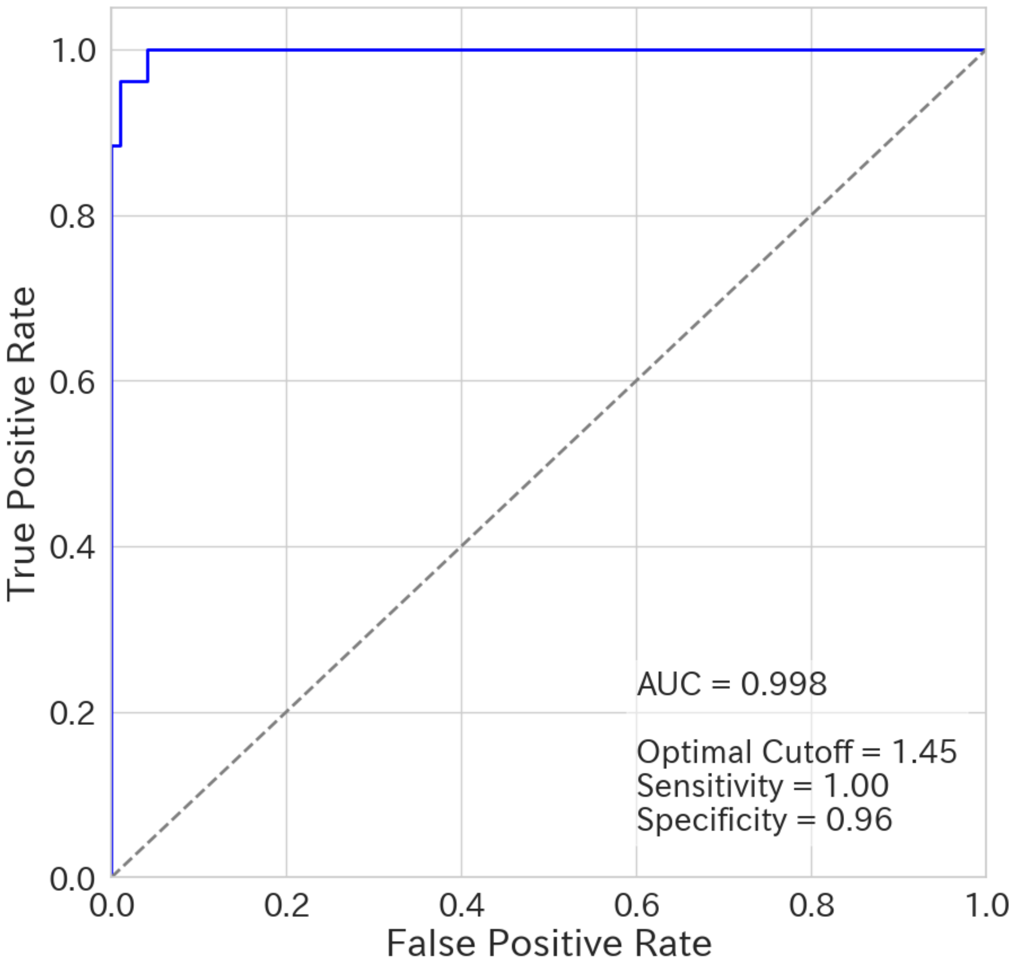

The area under the curve (AUC) was 0.998 (95% CI 0.991–1.000). The optimal cut-off (Youden's index) was 1.45 U/mL, yielding sensitivity 1.00 and specificity 0.96. UC cases included 26 older-onset patients (Japanese 19, U.S. 7), compared with 97 non-IBD controls. Due to the limited and imbalanced sample, results should be interpreted as exploratory.

Supplementary Table S1. Basic characteristics of the study population

Disease	Ethnicity	n (%)	Age			Sex n (%)		
			Median	IQR	p-values (US vs JP)	Female	Male	p-values (US vs JP)
**UC**	US	212 (47.0%)	43.0	(31.0, 58.0)	4.39e-01	107 (50.5%)	105 (49.5%)	3.48e-01
	JP	239 (53.0%)	44.0	(31.0, 55.0)		109 (45.6%)	130 (54.4%)	
	Total	451	43.0	(31.0, 57.0)		216 (47.9%)	235 (52.1%)	
**CD**	US	269 (47.5%)	46.0	(34.0, 61.0)	1.69e-05	135 (50.2%)	134 (49.8%)	5.63e-08
	JP	297 (52.5%)	42.0	(32.0, 50.0)		82 (27.6%)	215 (72.4%)	
	Total	566	44.0	(33.0, 55.0)		217 (38.3%)	349 (61.7%)	
**IBDU**	US	1 (5.3%)	71.0	(71.0, 71.0)	-	1 (100.0%)	0 (0.0%)	6.84e-01
	JP	18 (94.7%)	36.0	(29.75, 47.5)		5 (27.8%)	13 (72.2%)	
	Total	19	36.0	(30.5, 48.5)		6 (31.6%)	13 (68.4%)	
**Postoperative UC**	US	0 (0.0%)	-	-	-	-	-	-
	JP	5 (100.0%)	50.0	(47.0, 64.0)		3 (60.0%)	2 (40.0%)	
	Total	5	50.0	(47.0, 64.0)		3 (60.0%)	2 (40.0%)	
**Others**	US	7 (13.5%)	61.0	(43.5, 73.0)	9.04e-01	6 (85.7%)	1 (14.3%)	1.92e-01
	JP	45 (86.5%)	52.0	(42.0, 75.0)		23 (51.1%)	22 (48.9%)	
	Total	52	56.5	(41.75, 75.0)		29 (55.8%)	23 (44.2%)	
**Control**	US	25 (55.6%)	57.0	(47.0, 61.0)	5.81e-06	9 (36.0%)	16 (64.0%)	2.14e-01
	JP	20 (44.4%)	30.0	(30.0, 41.25)		3 (15.0%)	17 (85.0%)	
	Total	45	46.0	(30.0, 60.0)		12 (26.7%)	33 (73.3%)	
**Total**	US	514 (45.2%)	46.0	(33.0, 60.75)	1.01e-04	258 (50.2%)	256 (49.8%)	2.12e-06
	JP	624 (54.8%)	43.0	(31.0, 53.0)		225 (36.1%)	399 (63.9%)	
	Total	1138	44.0	(32.0, 57.0)		483 (42.4%)	655 (57.6%)	

UC, ulcerative colitis; CD, Crohn's disease; IBDU, inflammatory bowel disease unclassified; IQR, Interquartile range; US, U.S. cohort (self-reported White); JP, Japanese cohort

Supplementary Table S2. Breakdown of other intestinal diseases and positivity rates of anti-integrin αvβ6 antibody.

Disease	n	Positive	Negative	Positive rate
Intestinal Behçet's disease	9	0	9	0.0%
Ischemic colitis	12	0	12	0.0%
Microscopic colitis	7	1	6	14.3%
Diverticular bleeding	6	0	6	0.0%
Neuroendocrine tumors of colon and rectum	5	0	5	0.0%
Colorectal cancer	4	0	4	0.0%
Familial adenomatous polyposis	3	0	3	0.0%
Diverticulitis	2	0	2	0.0%
Hereditary non-polyposis colorectal cancer	1	0	1	0.0%
Eosinophilic gastroenteropathy	1	0	1	0.0%
Mesenteric panniculitis	1	0	1	0.0%
Cronkhite-Canada syndrome	1	0	1	0.0%

Supplementary Table S3. Titers and positivity rates of anti-integrin αvβ6 antibody.

Group	n	anti-αvβ6 level (U/ml)				Positivity rate			
		median [IQR]	p-value (vs Control)	q-value (FDR)	Effect size	n (%)	p-value (vs Control)	q-value (FDR)	OR (95%CI)
UC	451	20.2 [5.48,54.2]	1.29E-24	5.16E-24	0.460	385 (85.4)	3.75E-38	1.50E-37	525 (31.9-8628)
CD	566	0.624 [0.389,1.35]	2.83E-05	3.77E-05	0.169	93 (16.4)	6.19E-03	8.25E-03	17.7 (1.08-290)
IBDU	19	18.4 [1.36,82.8]	1.47E-08	2.94E-08	0.709	13 (68.4)	4.21E-09	8.42E-09	195 (10.2-3727)
Others	52	0.310 [0.221,0.457]	1.99E-01	1.99E-01	-0.131	1 (1.9)	1.00	1.00	1.76 (0.0578-53.9)
Control	45	0.400 [0.238,0.570]	-			0 (0)	-		-
(postoperative UC)	5	1.29 [1.21,23.4]	-		-	2 (40.0)	-		-

UC, ulcerative colitis; CD, Crohn’s disease; IBDU, inflammatory bowel disease unclassified; BD, intestinal Behçet's disease; IQR, Interquartile range; OR, odds ratio; CI, confidence interval

Supplementary Table S4. Association of anti-integrin αvβ6 antibody titer with disease sub-phenotypes

Phenotype	n	Titer of anti-αvβ6 (U/ml)			
		Coefficients	Median [IQR]	p-values	FDR
**Ulcerative colitis**					
Cohort				5.42e-02	0.126
U.S. (self-reported White)	212		19.1 [3.93:44.8]		
Japanese	239		23.0 [6.88:56.9]		
Sex				1.11e-01	0.194
Male	235		24.4 [6.00:51.0]		
Female	216		17.8 [4.61:54.4]		
Age	451	-1.08	20.2 [5.48:54.2]	**1.49e-02**	0.104
Age at diagnosis	418	-0.28	21.6 [5.91:55.2]	6.06e-01	0.601
Disease duration	418	-2.10	21.6 [5.91:55.2]	**3.22e-02**	0.113
Disease Extent				4.78e-01	0.558
E1 (proctitis)	27		14.7 [4.86:37.4]		
E2 (left sided colitis)	116		20.9 [6.76:46.7]		
E3 (pancolitis)	287		22.9 [5.63:57.9]		
Colectomy*				1.98e-01	0.277
Yes	6		50.2 [19.6:118]		
No	232		22.9 [6.92:56.5]		
**Crohn's disease**					
Cohort				**1.11e-02**	**0.0289**
U.S. (self-reported White)	269		0.785 [0.337:2.23]		
Japanese	297		0.559 [0.398: 0.965]		
Sex				7.46e-02	0.139
Male	349		0.604 [0.385:1.16]		
Female	217		0.703 [0.398: 1.85]		
Age		-0.029	0.624 [0.389:1.34]	7.58e-01	0.758
Age at diagnosis	551	0.122	0.624 [0.389:1.298]	2.85e-01	0.371
Disease Location				**5.09e-05**	**< 0.001**
L1 (ileal)	134		0.653 [0.398:1.00]		
L2 (colonic)	100		1.149 [0.491:12.5]		
L3 (ileocolonic)	332		0.582 [0.366:1.12]		
Upper gastrointestinal disease				5.11e-02	0.111
No	372		0.677 [0.384:1.80]		
Yes	132		0.569 [0.390:0.921]		
Small bowel involvement				**9.52e-06**	**<0.001**
No	100		1.15 [0.491:12.5]		
Yes	466		0.600 [0.377:1.10]		
Colonic involvement				1.26e-01	0.205
No	134		0.653[0.398:1.00]		
Yes	432		0.614 [0.385:1.54]		
Disease Behavior				**2.17e-03**	**0.0094**
B1	213		0.735 [0.403:3.61]		
B2	170		0.531 [0.360:0.949]		
B3	170		0.642 [0.389:1.23]		
Fistula				6.88e-01	0.745
No	384		0.614 [0.391:1.45]		
Yes	170		0.642 [0.390:1.23]		
Stenosis				**3.50e-03**	**0.0114**
No	251		0.697 [0.398:2.62]		
Yes	306		0.582 [0.378:1.08]		
Perianal disease				1.96e-01	0.283
No	320		0.657 [0.385:1.87]		
Yes	244		0.605 [0.390:1.05]		
Intestinal resection				5.54e-01	0.645
No	95		0.553 [0.370:0.945]		
Yes	200		0.564 [0.404:0.980]		

*Colectomy after serum sampling; IQR, interquartile range

Supplementary Table S5. Comparison of patient backgrounds and anti-αvβ6 by cohort

	Overall	U.S. cohort (self-reported White)	Japanese cohort	p-value	FDR
**Ulcerative Colitis**					
n	451	212	239		
Sex = Male (%)	235 (52.1)	105 (49.5)	130 (54.4)	3.48e-01	0.418
Age (median [IQR])	43.0 [31.0, 57.0]	43.0 [31.0, 58.0]	44.0 [31.0, 55.0]	4.39e-01	0.439
Age at diagnosis (median [IQR])	29.0 [20.0, 40.0]	26.0 [19.0, 37.0]	30.5 [21.0, 43.75]	**6.20e-03**	**0.186**
Extent (%)				**6.03e-03**	**0.186**
E1 (proctitis)	27 (6.3)	9 (4.7)	18 (7.5)		
E2 (left-sided colitis)	116 (27.0)	39 (20.4)	77 (32.2)		
E3 (pancolitis)	287 (66.7)	143 (74.9)	144 (60.3)		
anti-αvβ6 titer (U/ml)	20.2 [5.5,54.2]	19.1 [3.9,44.8]	23.0 [6.9,56.9]	5.42e-02	0.0813
anti-αvβ6 positivity rate (%)	385 (85.4)	173 (81.6)	212 (88.7)	**4.60e-02**	0.0813
**Crohn's Disease**					
n	566	269	297		
Sex = Male (%)	349 (61.7)	134 (49.8)	215 (72.4)	**5.63e-08**	**< 0.001**
Age (median [IQR])	44.0 [33.0,55.0]	46.0 [34.0,61.0]	42.0 [32.0,50.0]	**1.69e-05**	**< 0.001**
Age at diagnosis (median [IQR])	23.0 [18.0,33.0]	24.5 [17.0,37.0]	23.0 [18.0,29.0]	2.46e-01	0.308
Location (%)				**1.58e-14**	**< 0.001**
L1 (ileal)	134 (23.7)	101 (37.5)	33 (11.1)		
L2 (colonic)	100 (17.7)	52 (19.3)	48 (16.2)		
L3 (ileocolonic)	332 (58.7)	116 (43.1)	216 (72.7)		
Disease Behaviour				8.00e-01	0.800
B1	134 (23.7)	107 (39.9)	106 (37.2)		
B2	100 (17.7)	81 (30.2)	89 (31.2)		
B3	332 (58.7)	80 (29.9)	90 (31.6)		
Perianal disease (%)	244 (43.3)	76 (28.4)	168 (56.8)	**1.91e-11**	**< 0.001**
Fistula (%)	170 (30.7)	80 (29.9)	90 (31.5)	7.486e-01	0.800
Stenosis (%)	306 (54.9)	135 (50.4)	171 (59.2)	**4.56e-02**	0.0651
anti-αvβ6 titer (U/ml)	0.6 [0.4,1.3]	0.8 [0.3,2.2]	0.6 [0.4,1.0]	**1.11e-02**	**0.0185**
anti-αvβ6 positivity rate	93 (16.4)	63 (23.4)	30 (10.1)	**3.23e-05**	**< 0.001**

anti-αvβ6, anti-integrin αvβ6 antibody; IQR, interquartile range

Supplementary Table S6. Multivariate analysis for association of anti-integrin αvβ6 antibody with disease sub-phenotypes

Phenotype	Titer of anti-αvβ6 (U/ml)		Positivity of anti-αvβ6	
	coefficient (95%CI)	p-value	OR (95%CI)	p-value
**Ulcerative colitis**				
Cohort				
U.S. (self-reported White)	0.0683 (-32.4:32.5)	9.97e-01	0.93 (0.51:1.72)	8.28e-01
Japanese			(reference)	(reference)
Age	-0.635 (-1.72:0.446)	2.48e-01	0.98 (0.96:1.01)	1.53e-01
Disease duration	-1.64 (-3.29:0.019)	5.27e-02	0.96 (0.93:0.99)	**2.22e-03**
**Crohn's disease**				
Cohort				
U.S. (self-reported White)	**6.86 (0.653:13.1)**	**3.04e-02**	**6.86 (0.653:13.1)**	**4.84e-03**
Japanese	(reference)	(reference)	(reference)	(reference)
Disease Location				
L1 (ileal)	(reference)	(reference)	(reference)	(reference)
L2 (colonic)	**10.1 (0.411:19.7)**	**4.10e-02**	**8.89 (3.95:20.0)**	**1.28e-07**
L3 (ileocolonic)	5.23 (-2.50:13.0)	1.84e-01	**2.74 (1.27:5.90)**	**1.01e-02**
Upper gastrointestinal disease		-	0.53 (0.24:1.16)	1.14e-01
Disease Behavior				
B1	**(reference)**	**(reference)**	**(reference)**	
B2	-5.77 (-13.1:1.58)	1.23e-01	**0.43 (0.22:0.84)**	**1.41e-02**
B3	-4.90 (-12.3:2.51)	1.94e-01	**0.51 (0.26:0.98)**	**4.21e-02**
Perianal disease		-	0.60 (0.34:1.07)	8.20e-02

IQR, interquartile range; OR, odds ratio; CI, confidence interval

Supplementary Table S7. Phenotypes and two autoantibodies in Japanese patients with IBDU.

Case	Age	Sex	Group*	anti-αvβ6		anti-EPCR		Age at onset	UC-like lesions	Ileal lesions	longitudinal ulcer	Anal lesions	Granuloma	Extraintestinal Complications
				Titer		MFI								
IBDU-01	51	F	Group A	0.99	Negative	-4.3	Negative	51	pancolitis	ulcer, stenosis	ileum	None	None	None
IBDU-02	38	M	Group A	0.45	Negative	-53.6	Negative	17	S,R	None	S**	IAF	R	None
IBDU-03	36	M	Group B	122.8	Positive	309.4	Positive	28	Ce,A,T,D,S	None	None	None	None	Uveitis, pyoderma gangrenosum
IBDU-04	21	F	Group B	14.7	Positive	61.7	Positive	18	Ce,A	multiple erosions	None	None	None	None
IBDU-05	29	M	Group B	44.6	Positive	22.8	Positive	14	Segmental	multiple erosion, ulcers	None	IAF	None	Type 2 AIP
IBDU-06	39	M	Group D	1.43	Negative	36.7	Positive	37	T,D,S	None	None	None	None	None
IBDU-07	32	M	Group A	1.03	Negative	5.6	Negative	31	Ce,A,T,D,S	None	None	None	None	PSC
IBDU-08	35	F	Group C	316.5	Positive	16.0	Negative	23	Ce,A,T,D,S	None	None	None	None	PSC
IBDU-09	46	F	Group C	58.8	Positive	13.6	Negative	42	Ce,A	None	None	None	None	PSC
IBDU-10	24	M	Group C	27.5	Positive	7.5	Negative	21	Ce,A,T,D	None	None	None	None	PSC

*Based on the definition in Figure 6
**Appears 10 years after onset
Ce, Cecum; A, Ascending colon; T, Transverse colon; D, Descending colon; S, Sigmoid colon; R, rectum; IAF, Intractable anal fistula; AIP, Autoimmune pancreatitis; PSC, primary sclerosing cholangitis

Supplementary Table S8. Results of the ROC analysis for anti-αvβ6 antibodies

Prediction	Predictor	Ethnicity	AUC (95% CI)	p-value (DeLong's test)	Optimal model		
					Cutoff value	Sensitivity	Specificity
UC (vs non-IBD)	anti-αvβ6	US	0.94 (0.90-0.97)	8.38e-03	2.45	0.82	0.97
		JP	0.98 (0.97-0.99)		1.39	0.95	1.00
		US+JP	0.96 (0.95-0.98)	-	1.29	0.92	0.96
IBD (vs non-IBD)	anti-αvβ6	US	0.79 (0.73-0.84)	1.11e-01	0.59	0.75	0.81
		JP	0.84 (0.80-0.88)		1.02	0.57	0.97
		US+JP	0.82 (0.79-0.85)	-	0.48	0.79	0.74
UC (vs CD)	anti-αvβ6	US	0.83 (0.79-0.86)	4.33e-06	2.31	0.84	0.75
		JP	0.93 (0.91-0.95)		2.15	0.92	0.89
		US+JP	0.88 (0.86-0.90)	-	2.28	0.88	0.83
MES ≥ 1	anti-αvβ6	JP	0.74 (0.62-0.85)	-	27.74	0.59	0.85
PMS ≥ 3	anti-αvβ6	JP	0.68 (0.60-0.77)	-	24.85	0.76	0.60
UC (vs non-IBD)*	anti-EPCR	US+JP	0.95 (0.91-0.98)	4.56e-01	20.07	0.81	1.00
	anti-αvβ6		0.96 (0.93-0.99)		2.72	0.90	0.96
	combined		0.98 (0.95-1.00)	-	-	0.96	0.96

*Analysis limited to cases where data for both antibodies were available (n=118)

ROC, Receiver operating characteristic; AUC, area under the curve; UC, ulcerative colitis; CD, Crohn’s disease; IBD, inflammatory bowel disease; anti-αvβ6, anti-integrin αvβ6 antibody; US, U.S. cohort (self-reported White); JP, Japanese cohort

Supplementary Table S9. Diagnostic performance of anti-αvβ6 in older-onset UC (age at diagnosis ≥60 years) vs non-IBD

Group	n	Median (U/mL)	IQR (U/mL)	Positivity (%)	Statistical test	AUC (95% CI)	Optimal cut-off (U/mL)
**UC (Age ≥60 yrs)**	26	21.87	10.08 – 64.32	96.2	Mann–Whitney U $p = 7.78\times10^{-15}$; Fisher's exact $p = 8.06\times10^{-24}$	0.998 (0.991–1.000)	1.45 Sensitivity=1.00 Specificity=0.96
**non-IBD controls**	97	0.35	0.23 – 0.53	1.0			

Median titers (IQR), positivity rates, and statistical test results are shown. Mann–Whitney U test compared titers, and Fisher's exact test compared positivity rates. ROC analysis was performed to evaluate diagnostic accuracy, with the AUC, 95% CI, optimal cut-off, sensitivity, and specificity listed.
